# Cognitive impairment in Wilson's disease

**DOI:** 10.1590/S1980-57642009DN30100004

**Published:** 2009

**Authors:** Norberto Anizio Ferreira Frota, Paulo Caramelli, Egberto Reis Barbosa

**Affiliations:** 1 MD, General Hospital of Fortaleza and Department of Neurology, University of São Paulo School Medicine, São Paulo, SP, Brazil.; 2 MD, PhD, Behavioral and Cognitive Neurology Unit Faculty of Medicine, Federal University of Minas Gerais, Belo Horizonte, MG, Brazil.; 3 MD, PhD, Department of Neurology, University of São Paulo School Medicine, São Paulo, SP, Brazil.

**Keywords:** Wilson's disease, cognition, dementia

## Abstract

Wilson's disease (WD) or hepatolenticular degeneration is a rare, genetic and systemic disease, caused by a deficit in the metabolism of copper, leading to its accumulation in different organs, mainly the liver, followed by the central nervous system, especially the basal ganglia. When symptoms begin between the second and third decades of life, approximately 50% of the patients show neurological symptoms. Although dystonia and dysarthria are the most common neurological signs, cognitive changes have been reported since the first cases were described in 1912. Memory change is one of the most common impairments, but other cognitive changes have been reported, including dementia in untreated cases. In this article we review the cognitive changes in WD patients and the occurrence of dementia.

## Abstract (pt)

Doença de Wilson (DW) ou degeneração hepatolenticular é uma doença sistêmica genética rara, causada por um déficit no metabolismo do cobre, levando a sua acumulação in diferentes órgãos, principalmente o fígado, seguido pelo sistema nervoso central, especialmente os gânglios da base. Quando os sintomas iniciam entre a segunda e terceira décadas de vida, aproximadamente 50% dos pacientes apresentam sintomas neurológicos. Apesar de distonia e disartria serem as alterações neurológicas mais comuns, mudanças cognitivas tem sido relatadas desde os primeiros casos em 1912. Alteração de memória é o comprometimento mais comum, mas outras alterações foram descritas, incluindo demência em casos não tratados. Nesse artigo nós revisaremos as alterações cognitivas em pacientes com DW e a ocorrência de demência.

Wilson's disease (WD) or hepatolenticular degeneration is a rare genetic and multisystemic condition which affects mainly the liver, followed by the central nervous system (CNS), cornea and kidneys. Its incidence is 1–2 cases per 100,000 persons, with prevalence of 1:30,000 (homozygotes) and 1:100 to 1:2,000 (heterozygotes).^1^

The first description of a patient with this condition was reported by Friedrich Theodor von Frerichs in 1861.^2^ However, it was only after Alexander Kinnier Wilson described a series of cases in 1912 that the condition became well known. At the time, four young members of the same family were studied; the clinical features involved involuntary movement, spasticity, dysarthria, dysphagia and psychiatric symptoms with a fatal evolution which consisted, from a pathological perspective, of cirrhosis and softening of the lenticular nucleus.^3^ In the following year Rumpel linked this condition to copper.^4^ The locus of WD is now known to lie in the long arm of chromosome 13, responsible for encoding ATP7B expressed predominantly in the liver. ATP7B is responsible for transporting copper within cells so that it can be subsequently incorporated into ceruloplasmin and excreted through the bile. This is the main excretion pathway for copper. Thus, defects in this enzyme cause the copper to accumulate inside the hepatocyte cytoplasm and lead to later necrosis and release into the blood plasma. Subsequently, this metal accumulates elsewhere, such as in the basal ganglia and cornea.^5-8^ Over 250 types of mutations have been found to date, making clinical features heterogeneous. In Brazil, the most common mutations are found in loci 3402DelC and L708P.^9^

Where symptoms of WD appear in childhood, hepatic features are the most commonly occurring. When symptoms manifest between the second and third decades of life, approximately 50% of the patients show neurological symptoms. The most common of these, according to a study of a Brazilian sample, are: dysarthria (91%), abnormal gait (75%), *risus sardonicus* (72%), dystonia (69%), rigidity (66%), bradykinesia (58%), rest tremor (55%), dysphagia (50%), postural instability (49%), cerebellar alterations (28%), chorea (16%) and athetosis (14%).^10^

There are several structures or regions in the CNS which can be affected, including the cerebellum, thalamus and subcortical white matter, although the basal ganglia is predominantly affected.^11^

The medical treatment of Wilson disease's patients can be based on the use of chelating agents. These are capable of raising blood copper concentration and its renal excretion and this could explain why some patients may experience worsening of symptoms in the beginning of treatment. This initial toxicity is the reason why some authors prefer the use of zinc sulphate or acetate, which has a less aggressive profile in terms of collateral symptoms and is a copper lowering agent from the outset. Zinc treatment has been indicated ideally for medical treatment naïve individuals.^12^

Cognitive abnormalities, although acknowledged since the first description by Wilson, are under discussion to this day. Cummings cited the study conducted by Wilson as the first description of a case of subcortical dementia, a dementia pattern consisting of executive dysfunction, apathy and depression, as opposed to cortical dementias, in which aphasia, agnosia, apraxia and amnesia are predominant, such as in Alzheimer's disease.^13^ This kind of dementia has been associated to serum free copper. Patients with Alzheimer's disease and a high level of blood free copper have a worse outcome than patients with normal levels.^14^

Several studies have since tried to evaluate the cognitive abnormalities of WD, classifying it as a cause of reversible subcortical dementia. However, results are conflicting.

In this paper we discuss the cognitive abnormalities shown by patients suffering from WD and investigated whether they lead to functional impairment.

## Cognitive abnormalities

Cognitive deterioration in patients suffering from WD has been described since the first cases were reported in the early 20^th^ century.^3^ Patients showing neurological motor symptoms usually also present with, from the outset, changes in behavior or cognitive decline.^15^ This decline appears in approximately 25% of the patients.^16^ Depending on the test used and whether the patients present with more hepatic or neurological symptoms, this figure can reach up to 40%.^17^

Patients displaying exclusively hepatic symptoms tend to perform the same as normal voluntary participants on cognitive tests.^18,19^ Patients displaying neurological symptoms performance worse in comparison with control groups^18-21^ and with asymptomatic patients or those who have exclusively hepatic conditions. Only one study found slightly higher performance in executive functions when comparing patients with neurological manifestations against those with hepatic symptoms.^20^

Patients suffering from WD show a significantly poorer performance on global cognitive tests such as the Mini-Mental State Examination^17^ and the Mattis Dementia Rating Scale^18^ compared to healthy voluntary participants.^17,18^ Motor features may have influenced this impaired performance.^18^ Intelligence tests such as the WAIS^18,19^ and other similar instruments^21^ have shown conflicting results. One study found poorer performance on the WAIS test, restricted to the non-verbal part^18^ while another found performance to be worse in both verbal and non-verbal components.^19^ Other intelligence tests have revealed no statistical difference in relation to control groups.^21^ Even in those studies which have encountered differences in performance, these lay within the range of normality (low average).^18,19^

Memory deficits have been described by several authors.^18,19,22^ These authors have found that patients suffering from WD show lower capacity to both learn words and recall them across all stages of the Rey Auditory-Verbal Learning Test (RAVLT). They also observed a small difference in learning rate between the first and the second exposure to stimuli in this patient group.^19^ However, no difference was observed in the rate of loss of information between the last exposure and recall after interference, nor any difference in recognition.^19,22^ The results profile in this memory test resembled that of patients suffering from Huntington's disease which differs from the profile found in Alzheimer's dementia. Results are conflicting in non-verbal memory tests such as Benton's, and in computerized tests. Although Seniow^19^ encountered poorer performance in relation to control groups, Lang^21^ and Portala et al.^20^ observed no significant difference. These studies with negative findings pooled all patients with WD into a single group regardless of the clinical features (neurological, hepatic or asymptomatic). This may have contributed to the negative findings.

Tests which evaluate executive functions such as Raven's Progressive Matrices, also show poorer performance by patients with WD compared with healthy controls. Tests which also evaluate reasoning have shown a difference between patients displaying neurological symptoms and control groups.^18,20,21^ No differences have been observed compared with performance of control groups on semantic verbal fluency (animals)^18^ although performance was significantly worst in phonemic fluency.^23^ The study by Lang also observed a difference in phonemic fluency between the two groups, although the author did not consider this result to be clinically significant.^21^ Language tests such as the Boston Naming Test have not shown any difference between the two groups.^18^ These findings suggest that the difference in performance between the two types of fluency might be more strongly attributable to dysfunction in frontal-subcortical circuits than to a language problem or dysarthria. Similar findings were found in Friedreich's ataxia.^23^ This difference in fluency has previously been described as a differential between Alzheimer's disease and frontotemporal dementia.^24^

Other tests which evaluate this circuit, such as forward and backward digit span, have shown no differences between WD patients and controls,^18,21^ although it did display abnormal results in another evaluation.^19^ The performance on the Wisconsin Card Sorting Test has also been described not to differ between patients and controls, revealing a similar number of responses and perseverant errors.^18^ Evaluations carried out using computerized tests have shown lower performance in digit span and divided attention tests, with an increase in perseverating responses.^21^ An increase in response time for both visual and auditory stimuli, as well as in perception speed was evident.^20,21^ In spite of these findings, no greater difficulty in information processing was observed.^25^

The summary of cognitive changes described in WD patients is depicted in Table 1.

**Table 1:** Tests results in WD.

Cognitive domain	Author	N	Patient scores	Control scores	*p*
Global assessment					
MMSE	Sinha et al.	34	24	-	-
DRS	Medalia et al.	19	139.26	142.2	0.014
WAIS - VIQ	Medalia et al.	19	99.0	106.87	0.153
WAIS - PIQ	Medalia et al.	19	91.79	104.93	0.008
WAIS - FIQ	Medalia et al.	19	95.53	106.4	0.047
WAIS - VIQ	Seniow et al.	50	97.9	112.76	<0.001
WAIS - PIQ	Seniow et al.	50	98.52	115.22	<0.001
WAIS - FIQ	Seniow et al.	50	98.83	115.12	<0.001
LPS - reasoning	Lang et al.	17	21.71	27.0	0.009*
Memory					
RAVLT	Glaberman et al.	19			<0.001
RAVLT	Seniow et al.	50			<0.0001
Benton	Seniow et al.	50			<0.0001
Benton	Lang et al.	17	13.18	13.18	0.438
WMS	Medalia et al.	19	101.21	114.20	<0.01
Executive functions					
Wisconsin Card Sorting categories	Medalia et al.	19	5.69	6	0.632
Wisconsin Card Sorting perseverations	Medalia et al.	19	10.16	5.06	0.164
Raven	Lang et al.	17	42.06	49.94	0.014*
Raven	Seniow et al.	50			<0.0001
Digit Span	Seniow et al.	50	5.43	7.0	<0.05
Digit Span	Lang et al.	17	10.65	10.53	0.413
Verbal Fluency FAS	Glaberman et al.	19			<0.01
Verbal Fluency Letter	Lang et al.	17	32.41	40.06	0.014*
Verbal Fluency Animals	Medalia et al.	19	20.06	21.94	0.24
Trail Making A	Medalia et al.	19	44.2	28	0.014
Trail Making A - errors	Medalia et al.	19	0.05	0.06	0.25
Trail Making B	Medalia et al.	19	86.4	58.1	0.018
Trail Making B - errors	Medalia et al.	19	0.26	0.33	0.25
Language					
Boston	Medalia et al.		79.36	80.73	0.114
Object naming	Lang et al.	1917	15.0	14.94	0.359
Visuo spatial					
Intelligence Structure - mental figure	Lang et al.	17	10.76	10.41	0.049*
Intelligence Structure - mental rotation	Lang et al.	17	8.59	10.35	0.028*
Perceptual Speed					
Perceptual Speed	Lang et al.	17	23.76	17.53	0.0025
Perceptual Maze Test	Portala et al.	19	-	-	<0.05

MMSE, Mini Mental State Examination; DRS, Dementia Rating Scale; WAIS, Wechsler Adult Intelligence Scale; VIQ, Verbal IQ; PIQ, Performance IQ; FIQ, Full-scale IQ; LPS, Achievement Assessment System; RAVLT, Ray Auditory Verbal Learning Test; WMS, Wechsler Memory Scale.

* The author used alpha adjustment with *p*<0.00263.

## Cognitive abnormalities and clinical features/imaging

Initially, abnormalities in memory and some other cognitive tests were associated with the impact of the motor features on such patients.^18^ However, following evaluation of the motor features by means of scales, no relationship has been observed between the degree of motor impairment and performance on the WAIS. Similarly, no correlation has been found between the psychiatric symptoms and cognitive features. These three domains of impairment seem to act independently.^26^

Patients who have had the disease for a longer period show poorer performance on visuospatial tests while older subjects show impaired performance on executive function tests. Patients who have an earlier onset also present poorer visuospatial performance and take longer to carry out motor activities.^20^ No correlation has been found between the motor or cognitive features and copper levels or ceruloplasmin.^17^

Evaluation of patients displaying both neurological symptoms and cranial computed tomography (CT) scan abnormalities revealed poorer performance on digit arrangement tests than patients showing no CT abnormalities. However, the author did not specify the nature of the abnormalities found.^21^

Patients displaying WD neurological symptoms with lesions confined to the basal ganglia were compared with patients displaying additional lesions in other areas using brain magnetic resonance imaging (MRI) exams. It was evident that patients who had lesions restricted to the basal ganglia also showed lower performance to that of control groups across all tests, although with less statistical significance when evaluated together with the patients who had additional lesions in other areas. Comparing both groups of neurological patients, there was a tendency toward better IQ performance and execution, albeit statistically insignificant, and with regard to comprehension, digit span, object assembly, block design and digit symbol subscores, better performance was seen by patients who had lesions limited to basal ganglia. However there was no difference between Rey's and Benton's memory tests, nor Raven's matrices, which suggests that even if the condition were restricted to the basal ganglia it can also lead to cognitive abnormalities.^19^

Although two studies have already made clear that functional SPECT exams can be more sensitive than MRI for diagnosing abnormalities in patients suffering from WD,^27,28^ such exams can show abnormalities even in patients who have hepatic symptoms only. No study has yet compared these abnormalities with motor clinical features or cognitive abnormalities.

## Dementia in Wilson's disease

The accounts produced by Wilson^3^ in the early 20^th^ century described, together with the motor symptoms of the condition, neuropsychiatric abnormalities which caused functional impairment in these patients. These features led Cummings to refer to WD as an etiology of dementia of the subcortical type.^13^

Several clinical accounts have since described cases of WD which showed cognitive abnormalities leading to functional impairment, corresponding to clinical features that resemble a dementia syndrome.^3,29,30^ All these studies demonstrated motor and cognitive improvement after initiating clinical treatment for WD. This observation led to WD being categorized as a reversible dementia, both after clinical treatment^29,30^ or after hepatic transplant.^31^ Cognitive abnormalities may persist even after treatment, as discussed previously.

Performance by WD patients on the several tests discussed earlier, although inferior to that of control groups, has proven to be mostly within normal levels (on average within 1 standard deviation).^18,19,21^ The fact that components such as language, semantic verbal fluency and performance on the Wisconsin Card Sorting Test were preserved has prevented some authors from considering WD as a dementia syndrome.^18^ Another point raised was that the majority of patients were ambulatory and independent with regard to their daily and routine activities,^21^ although none of the studies evaluated functional performance.

Systematic studies involving a greater number of WD patients have excluded subjects whose neurological state was more severely impaired. This might have contributed to the negative finding of dementia in WD for treated patients.^19^ A recent Brazilian study evaluating a large number of cases, including subjects manifesting various levels of neurological impairment, found a prevalence of dementia of 5% among these patients.^10^

Criteria for dementia diagnosis applied when most of the studies were carried out, considered memory impairment and progressive clinical decline to be necessary to reach this diagnosis. Nowadays, it is known that a static pattern for cognitive deficits is sufficient, and that memory impairment involves the learning domain and not only delayed recall. The diagnosis of some types of dementia is now even dismissing the mandatory presence of memory impairment, as is the case for dementia with Lewy bodies,^32^ dementia associated with Parkinson's disease^33^ and frontotemporal lobar degeneration.^34^ Thus, the lack of WD patients diagnosed with dementia even after clinical treatment could be the result of underestimated data. Regardless of this, it is already known that early treatment has an important impact on the course of WD.^35^

## Conclusion

WD is a rare neurological condition associated not only to motor impairment but also to cognitive abnormalities that can be severe in the initial stages if not treated and that remain, albeit in mild form, after commencing treatment. These abnormalities occur mainly with regard to attention, executive functions and memory (encoding), but may also appear even when only the basal ganglia are affected. Early diagnosis and treatment are crucial for better prognosis.
